# Spindle Assembly Checkpoint Acquisition at the Mid-Blastula Transition

**DOI:** 10.1371/journal.pone.0119285

**Published:** 2015-03-05

**Authors:** Maomao Zhang, Priyanka Kothari, Michael A. Lampson

**Affiliations:** 1 Department of Biology, University of Pennsylvania, Philadelphia, Pennsylvania, United States of America; 2 Cell and Molecular Biology Graduate Group, University of Pennsylvania, Philadelphia, Pennsylvania, United States of America; University of Virginia, UNITED STATES

## Abstract

The spindle assembly checkpoint (SAC) maintains the fidelity of chromosome segregation during mitosis. Nonpathogenic cells lacking the SAC are typically only found in cleavage stage metazoan embryos, which do not acquire functional checkpoints until the mid-blastula transition (MBT). It is unclear how proper SAC function is acquired at the MBT, though several models exist. First, SAC acquisition could rely on transcriptional activity, which increases dramatically at the MBT. Embryogenesis prior to the MBT relies primarily on maternally loaded transcripts, and if SAC signaling components are not maternally supplied, the SAC would depend on zygotic transcription at the MBT. Second, checkpoint acquisition could depend on the Chk1 kinase, which is activated at the MBT to elongate cell cycles and is required for the SAC in somatic cells. Third, SAC function could depend on a threshold nuclear to cytoplasmic (N:C) ratio, which increases during pre-MBT cleavage cycles and dictates several MBT events like zygotic transcription and cell cycle remodeling. Finally, the SAC could by regulated by a timer mechanism that coincides with other MBT events but is independent of them. Using zebrafish embryos we show that SAC acquisition at the MBT is independent of zygotic transcription, indicating that the checkpoint program is maternally supplied. Additionally, by precociously lengthening cleavage cycles with exogenous Chk1 activity, we show that cell cycle lengthening and Chk1 activity are not sufficient for SAC acquisition. Furthermore, we find that SAC acquisition can be uncoupled from the N:C ratio. Together, our findings indicate that SAC acquisition is regulated by a maternally programmed developmental timer.

## Introduction

The spindle assembly checkpoint (SAC) ensures that sister chromatids are correctly attached to spindle microtubules before anaphase onset. In the presence of unattached kinetochores, the SAC is active and inhibits the anaphase promoting complex/cyclosome [1]. This checkpoint maintains genomic integrity by preventing chromosome segregation errors.

Intriguingly, newly fertilized embryos of most metazoans lack SAC function. Immediately following fertilization, *Xenopus* embryos undergo metasynchronous cleavage divisions that cause surface contraction waves on the embryo, which are easily visualized. When spindle assembly is inhibited by microtubule depolymerizing agents like colchicine and vinblastine, embryos continue to have periodic surface contraction waves, indicating that cell cycle progression is not affected [2,3]. Furthermore, Maturation Promoting Factor (MPF) activity continues to oscillate in embryos after microtubule depolymerization, providing further evidence for lack of a spindle checkpoint [4].

In *Xenopus* and zebrafish, cell cycles elongate dramatically and are extensively remodeled at the mid-blastula transition (MBT): rather than the rapid replication-mitosis cycles typical of cleavage divisions, cells acquire gap phases [5] and cell cycle checkpoints. When treated with DNA damaging agents or spindle poisons, post-MBT embryos arrest their cell cycles similarly to somatic cells [6–8]. Furthermore, the MBT marks a period of robust transcriptional activity, when developmental control switches from maternal to zygotic [9–11]. The simultaneous appearance of multiple changes at the MBT makes it difficult to determine which may control SAC acquisition, and we considered several possible models.

First, SAC function at the MBT could be under either maternal or zygotic control. In oviparous organisms where embryogenesis occurs outside the mother, embryos rely on maternal transcripts loaded during oogenesis to drive many early developmental events after fertilization. Therefore, the SAC may not function in pre-MBT embryos simply because checkpoint components are not maternally supplied and depend on zygotic transcription, which is induced robustly at the MBT.

Second, Chk1 kinase activity could promote SAC function at the MBT. Chk1 kinase is a regulator of cell cycle progression that is well known for its role in the DNA damage checkpoint. Upon activation after DNA damage, Chk1 phosphorylates multiple substrates to promote cell cycle delay [12]. However, Chk1 also plays an important role at the MBT. In *Xenopus*, Chk1 is transiently activated at the MBT and targets Cdc25 phosphatase for degradation, inducing cell cycle elongation [13]. Moreover, *Drosophila* embryos with a mutation in *grapes*, the Chk1 homolog, do not lengthen their cell cycles at the MBT, undergoing two additional syncytial pre-MBT-like divisions [14]. Chk1 is also required for the SAC in somatic cells [15–17], which suggests that Chk1 activation at the MBT could lead to SAC acquisition.

Third, many MBT events are governed by the nuclear-to-cytoplasmic ratio (N:C ratio). Because cleavage-stage embryos divide without cell growth, cell volumes halve at each division until a threshold N:C ratio is achieved at the MBT [9,18]. Several MBT events, such as transcriptional activation and DNA damage checkpoint acquisition, occur prematurely if the N:C ratio is precociously increased in embryos [9,19].

Alternatively, SAC function could be regulated by a cell cycle-independent timer mechanism, uncoupled from the N:C ratio, that begins at fertilization or egg activation. Several MBT events seem to be controlled temporally [20]. For example, degradation of cyclins A and E1 in *Xenopus* embryos contributes to cell cycle lengthening at the MBT and is independent of the N:C ratio and zygotic transcription [21,22].

Using zebrafish embryos, which are easily manipulated and amenable to fixed and live cell imaging, we investigated the influence of large-scale changes that occur at the MBT on SAC acquisition. We demonstrate that the SAC does not rely on transcriptional activity. We also show that Chk1 activity and cell cycle elongation before the MBT are not sufficient for precocious checkpoint function, and that SAC acquisition does not depend on a threshold N:C ratio. We conclude that while occurring concomitantly with cell cycle remodeling and an increase in zygotic transcription, SAC function is independently regulated by a developmental timer.

## Results

### Pre-MBT Zebrafish embryos do not delay mitosis after microtubule disruption

**Fig 1:**
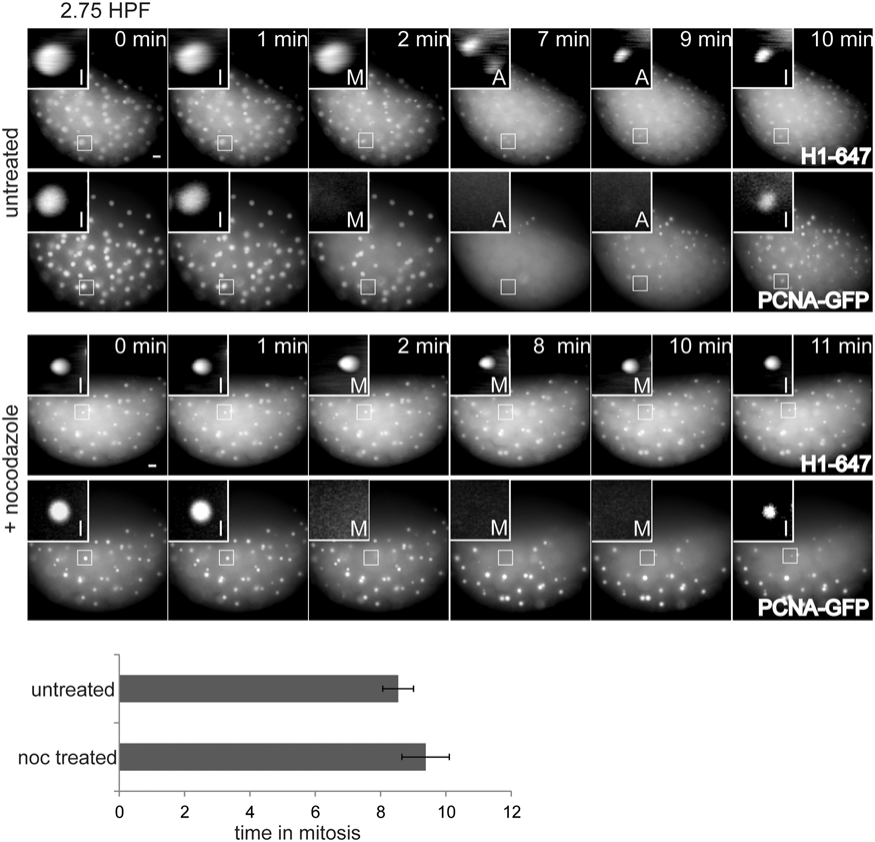
Pre-MBT embryos lack a functional SAC. Embryos were injected with Alexa 647-Histone H1 and PCNA-GFP proteins, treated with or without nocodazole before the MBT at 2 HPF, then imaged live. Images show cell cycle progression based on Histone H1 and PCNA from an embryo at 2.75 HPF. Insets are displayed with higher contrast settings to show the changing morphology of a single nucleus at different cell cycle stages: I, interphase; M, prometaphase/metaphase; A, anaphase. In the montage, time between metaphase and the next interphase is 8 min for untreated control embryos and 9 min for nocodazole-treated embryos. Graph shows average length of mitosis for each condition (n≥14 embryos for each, from three independent experiments). Error bars indicate S.D.; P>0.05; two-tailed Student’s t test; scale bars 20 μm.

The SAC delays mitosis in response to microtubule disruption, which we used to test for SAC function in early embryos. To determine the time in mitosis, we used a live-cell imaging assay to monitor nuclear localization of Proliferating cell nuclear antigen (PCNA) during the cleavage cycles in zebrafish embryos. PCNA is a replication factor that localizes to the nucleus soon after anaphase, when the nuclear envelope is reformed. It remains in the nucleus throughout S-phase, and then disperses into the cytoplasm when the nuclear envelope breaks down during prometaphase [23]. We injected GFP-tagged PCNA protein into 1-cell stage embryos and measured the times of nuclear envelope breakdown and reformation for successive cell cycles (Fig. 1 and S1–S4 Movies). Embryos were co-injected with fluorescently labeled Histone H1 protein to serve as a chromatin marker during M-phase when the PCNA-GFP is diffuse throughout the cytoplasm.

To test for a SAC response, pre-MBT embryos were treated with nocodazole. Upon drug treatment, cells formed compact nuclei and were unable to separate their chromosomes or complete cytokinesis (Fig. 1). Despite these catastrophic failures, PCNA-GFP continued to localize to and disperse from nuclei, indicating nuclear envelope breakdown and reformation. Importantly, the total cell cycle time (data not shown) and length of mitosis were unchanged (Fig. 1), demonstrating lack of a SAC in cleavage-stage embryos, consistent with previous findings [2–4].

### SAC acquisition at the MBT is a maternal program

To distinguish whether SAC acquisition is governed by a maternal or zygotic program, we inhibited transcriptional activity by injecting one-cell stage embryos with α-amanitin, an inhibitor of RNA polymerase II [24,25], which inhibits transcription until 4 HPF (hours post fertilization) [8]. After α-amanitin treatment embryos arrested at the sphere stage as expected [24]. To test for a mitotic checkpoint response in the absence of zygotic transcription, embryos were injected with α-amanitin at the 1-cell stage, then treated with nocodazole at 3.25 HPF, when the MBT has already occurred and the SAC is functional in control embryos.

**Fig 2:**
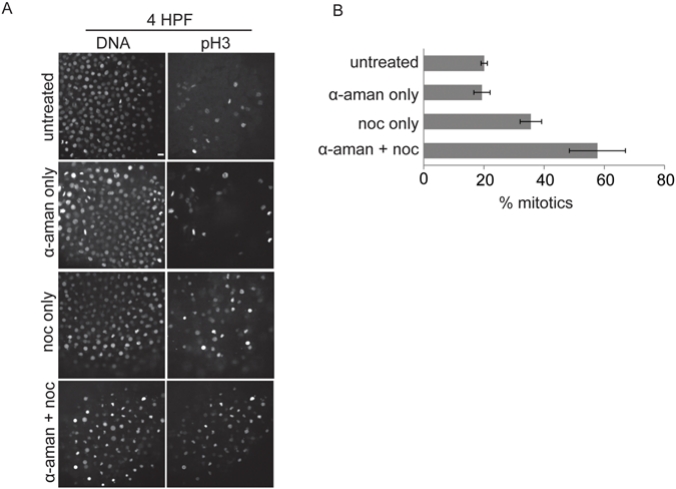
SAC acquisition does not rely on zygotic transcription. 1-cell stage embryos were injected with α-amanitin as indicated, treated with or without nocodazole at 3.25 HPF for 45 min, fixed at 4 HPF, and stained for pH3 and DNA. Representative images are shown (A). The percent of nuclei positive for pH3 was calculated and averaged over multiple embryos (B, n≥18 for each condition). Error bars indicate s.e.m, calculated over three independent experiments; scale bar 20 μm.

Cell density, motility, and cell cycle asynchrony limit our ability to measure mitotic timing live in post-MBT embryos. Instead, to assay for SAC function after nocodazole treatment, embryos were fixed and stained for phosphorylated Serine 10 on Histone H3 (pH3), a well-established marker for mitosis [26]. The accumulation of pH3-positive cells serves as a readout for cells arrested in mitosis. The mitotic index increased in control post-MBT embryos treated with nocodazole, indicating a functional SAC (Fig. 2). Inhibition of zygotic transcription with α-amanitin did not change the mitotic index of post-MBT embryos and did not prevent cell cycle arrest in response to nocodazole after the MBT: cells still accumulated in mitosis after nocodazole treatment (Fig. 2). These data demonstrate that transcription is unnecessary for SAC acquisition at the MBT. Rather, SAC components are maternally supplied but are not functional until the MBT. We also note that α-amanitin increased the mitotic index in the presence of nocodazole, which raises the possibility that zygotic genes may contribute to mitotic exit when the checkpoint has not been satisfied (i.e., mitotic slippage).

### Precocious Chk1 activity and cell cycle elongation are not sufficient for SAC acquisition

**Fig 3:**
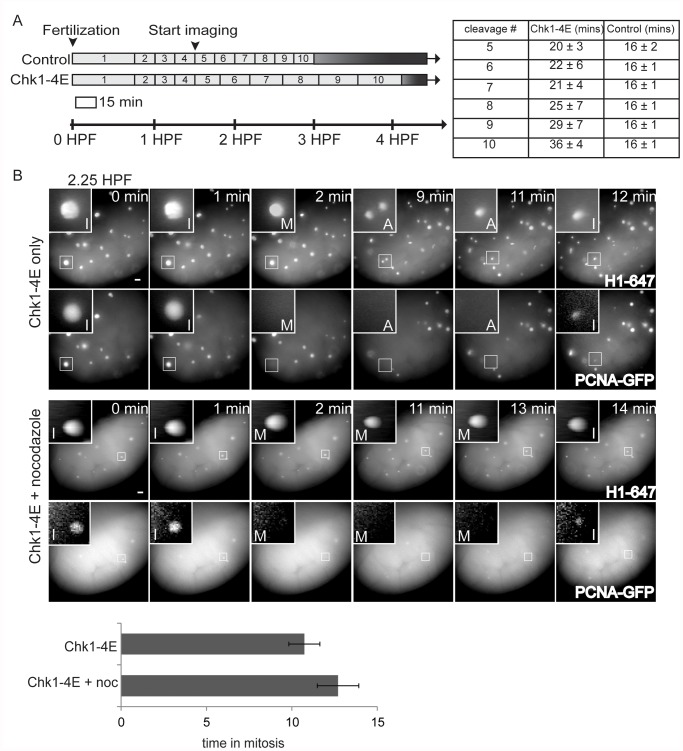
Precocious Chk1 activity and cell cycle elongation are not sufficient for SAC acquisition. (A) Schematic of cell cycle lengths for the first 10 cleavage divisions of embryos injected with Alexa 647-Histone H1, with or without Chk1-4E-GFP mRNA. Cell cycles lengths were measured as time between metaphases, starting from 1.5 HPF, based on Histone H1 morphology. Embryos injected with H1-647 alone (control) had consistent cleavage divisions that each lasted ~16 min as expected [5] while embryos injected with Chk1-4E mRNA and H1-647 had cleavage cycles that progressively lengthened. The measured cell cycle lengths for cleavages 5–10 are indicated by the length of each bar in the schematic and shown in the table (n >4 embryos; error indicates S.D.) (B) Embryos were injected with Alexa 647-Histone H1 and PCNA-GFP proteins and Chk1-4E mRNA, treated with our without nocodazole at 2.25 HPF, then imaged live until 3 HPF. Images show cell cycle progression based on Histone H1 and PCNA. Insets are displayed with higher contrast settings to show nuclear morphology as in Fig. 1. In the montage, time between metaphase and the next interphase is 10 min without nocodazole and 12 min for nocodazole-treated embryos. There was no measurable progressive lengthening of mitoses during the cleavage divisions, regardless of drug treatment. Graph shows average length of mitosis for each condition (n≥12 for each condition, from three independent experiments). Error bars indicate S.D.; P>0.05; two-tailed Student’s t test; scale bars 20 μm.

Given the activation of Chk1 at the MBT and its role in the SAC, we hypothesized that Chk1 activity could control SAC acquisition at the MBT. To test the effect of precocious Chk1 activity on SAC function prior to the MBT, embryos were injected with an mRNA encoding an active, phosphomimetic Chk1 mutant (Chk1-4E) [8,27], together with fluorescently labeled PCNA and Histone H1 proteins. Total cell cycle times and time spent in mitosis were then measured live with our PCNA-GFP localization assay. Exogenous Chk1-4E progressively lengthens cell cycles in pre-MBT embryos starting from the 5–6^th^ cleavage cycle at ~2 HPF (Fig. 3A), consistent with the known function of Chk1 in inhibiting Cdc25 activity [12]. When pre-MBT embryos expressing Chk1-4E are treated with nocodazole at 2.25 HPF, PCNA-GFP nuclear localization and dispersion is unperturbed, and there is no increase in the duration of mitosis (Fig. 3B). Though previous work showed that Chk1 activity is necessary for MBT cell cycle remodeling [13,14], our data demonstrate that precocious Chk1 activity and cell cycle elongation are not sufficient to mount a SAC response prior to the MBT.

### SAC acquisition depends on a developmental timer

The N:C ratio increases more slowly in Chk1-4E injected embryos compared to control embryos due to their elongated cell cycles, which allowed us to test whether SAC activation is coupled to the N:C ratio or to a developmental timer. Control embryos complete 10 cleavages at 3 HPF, which marks the onset of the MBT. However, embryos injected with Chk1-4E mRNA complete only 8 cleavages by 3 HPF (Fig. 3A). If SAC function requires that embryos obtain the threshold N:C ratio achieved after 10 cleavages, we would not expect SAC acquisition in Chk1-4E embryos until they reach this threshold N:C ratio. In contrast, if SAC acquisition is controlled by a developmental timer that is independent of the N:C ratio, we expect SAC function in Chk1-4E embryos at a lower N:C ratio, at ~ 3 HPF as in control embryos.

**Fig 4:**
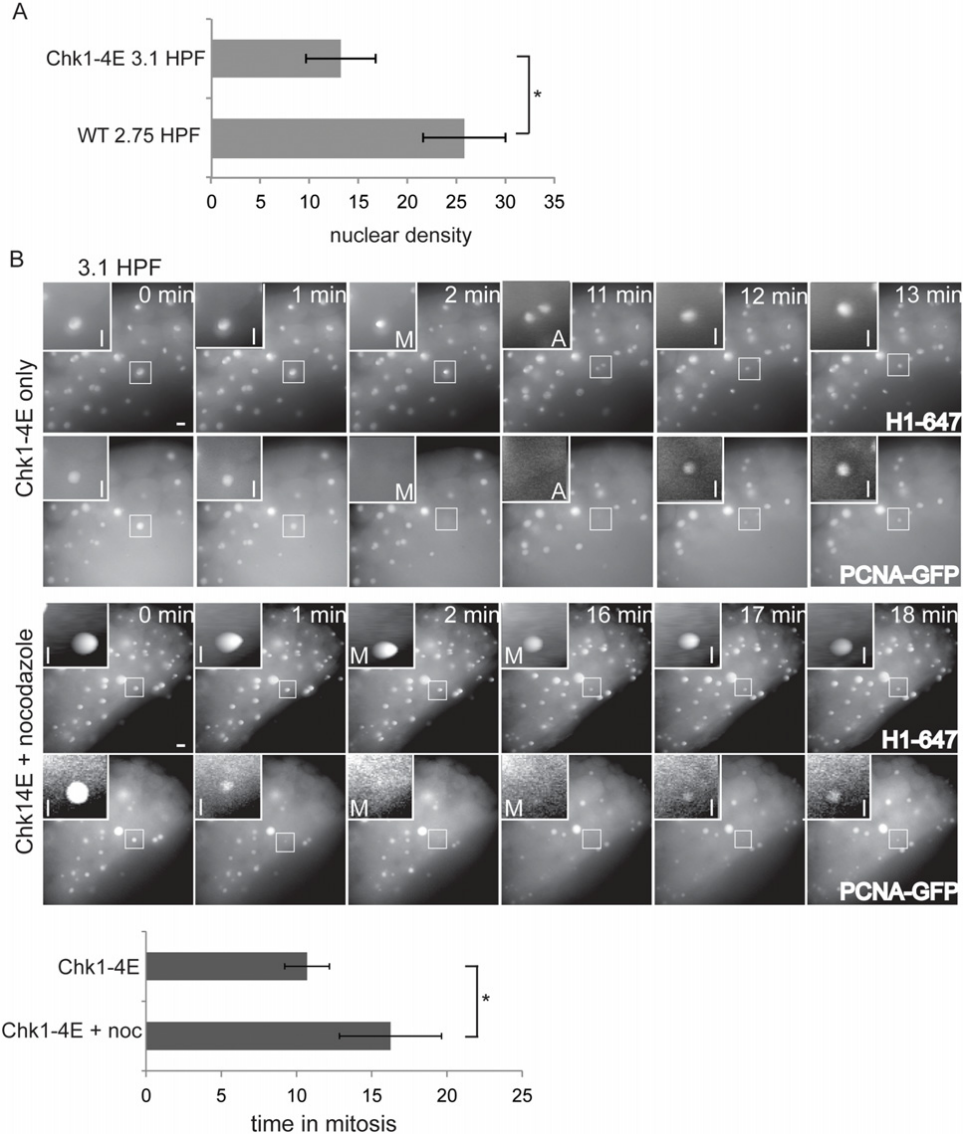
SAC acquisition is independent of the N:C ratio. (A) Nuclear density was measured for embryos injected with Alexa 647-Histone H1 and PCNA-GFP proteins and Chk1-4E mRNA at 3.1 HPF, or embryos injected only with H1-647 and PCNA-GFP proteins at 2.75 HPF. Error bars are S.D.; n≥11; P≤0.001. (B) Embryos were injected with Alexa 647-Histone H1 and PCNA-GFP proteins and Chk1-4E mRNA, treated with or without nocodazole at 3.1 HPF, then imaged live. Images show cell cycle progression based on Histone H1 and PCNA-GFP. Insets are displayed with higher contrast settings to show nuclear morphology as in Fig. 1. In the montage, time between metaphase and the next interphase is 10 min without nocodazole and 16 min for nocodazole-treated embryos. Graph shows average length of mitosis for each condition (n≥13 from four independent experiments). Error bars indicate S.D.; * P≤ 0.001; two-tailed Student’s t test; scale bars 20 μm.

To distinguish between the timer and N:C ratio models, we compared control embryos at 2.75 HPF, before the MBT, to Chk1-4E embryos just past 3 HPF. To compare N:C ratios, we measured the density of nuclei in the embryo, which increases with each cleavage as the total volume of the embryo remains constant. The nuclear density in Chk1-4E embryos at 3.1 HPF is much lower than the nuclear density in control embryos at 2.75 HPF (Fig. 4A, and compare nuclear densities in Fig. 1 vs Fig. 4). Whereas control embryos at 2.75 HPF lack SAC function (Fig. 1), Chk1-4E embryos at 3.1 HPF spend significantly longer in mitosis (16.3 vs. 10.7 min) after nocodazole treatment (Fig. 4B), indicating that the SAC is functional. Thus, Chk1-4E embryos have a functional SAC at 3.1 HPF, at an N:C ratio that is lower than that of control pre-MBT embryos at 2.75 HPF, which do not have a SAC. These data indicate that SAC function depends on a developmental timer rather than a threshold N:C ratio.

## Discussion

Our findings provide insight into how the early embryo acquires SAC function. Specifically, we investigated whether SAC acquisition is coordinated with other major developmental changes during the MBT, including cell cycle elongation and Chk1 activity, a threshold N:C ratio, and zygotic transcription. Despite the coincident appearance of these events in the embryo, we show that SAC function can be uncoupled from other MBT events.

We first investigated whether SAC acquisition is coupled with transcriptional activity, as past studies have suggested that zygotic transcription and certain aspects of cell cycle remodeling are coordinated. For example, addition of the G2 phase of the cell cycle during MBT cell cycle remodeling in zebrafish relies on zygotic transcription [24]. Conversely, cell cycle elongation may be required for zygotic transcription, as transcripts are often aborted in rapid cell cycles in *Drosophila* due to time constraints [28]. Despite the clear co-regulation of cell cycle remodeling and transcriptional activity at the MBT, we show that zygotic transcription is not required for SAC acquisition, implying that SAC components are maternally loaded. Similarly, our previous work shows that DNA damage checkpoint acquisition occurs independent of transcriptional activity[8].

Our data are consistent with similar findings in *Xenopus*, which showed that blocking transcription after the 8-cell stage in dissociated blastomeres does not prevent SAC acquisition [29]. However, gene expression profiling has revealed that many zygotic genes are expressed during the cleavage stages, some as early as the 4-cell stage [30]. Thus, the previous experiments did not fully account for possible early zygotic transcription of SAC components, which may provide a sufficient pool of mRNA for SAC protein synthesis and accumulation. In contrast, we inhibited transcription immediately after fertilization, at the 1-cell stage, ruling out the possibility of a zygotic contribution of SAC components.

We also investigated the role of the N:C ratio in SAC acquisition. The coordination of many MBT events seems to stem from the N:C ratio, which increases with every cleavage cycle. For example, the replication factors which account for the fast S-phase in pre-MBT embryos are titrated as the N:C ratio increases, leading to slowed replication at the MBT and increased interphase duration [31]. Additionally, the N:C ratio can affect DNA damage checkpoint acquisition: addition of exogenous DNA to pre-MBT Xenopus embryos, to mimic the N:C ratio typical of the MBT, can lead to precocious checkpoint function after DNA damage [32,33].

By precociously increasing cell cycle lengths in pre-MBT embryos with Chk1, we show that cell cycle lengthening and Chk1 activity are not sufficient for SAC acquisition. Furthermore, lengthening pre-MBT cell cycles slows the increase in N:C ratio, and we find that SAC acquisition does not depend on a threshold N:C ratio. This result is consistent with previous experiments which showed that individual blastomeres isolated from dissociated *Xenopus* embryos acquire a functional SAC with varying N:C ratios [29,34]. Our results indicate that SAC acquisition is regulated by a timer mechanism that does not rely on Chk1 activity or zygotic transcription.

Although we find that a threshold N:C ratio is not necessary for SAC acquisition, the SAC can become functional prematurely in *Xenopus* egg extracts if sperm nuclei are added to increase the N:C ratio to a threshold level [35]. Together these findings suggest that both a developmental timer and increases in the N:C ratio can contribute to SAC regulation. Increasing N:C ratio may artificially titrate as-yet unidentified cytosolic SAC inhibitors that are present during the cleavage stages, or increased numbers of kinetochores may enhance production of a SAC signal. During normal development, when the number of kinetochores is fixed, a set time may be required for the synthesis and accumulation of SAC proteins that amplify signaling downstream from initial SAC activation at kinetochores. However, the need for accumulation of these proteins could be bypassed if large numbers of kinetochores amplify SAC signaling.

Our findings raise the question of how a maternally-controlled developmental timer regulates SAC acquisition at the MBT. Time could be required for either accumulation of SAC proteins from maternally supplied transcripts or degradation of a SAC inhibitor. For example, SAC function depends on multiple checkpoint proteins that are recruited to unattached kinetochores to generate the inhibitory signal that prevents anaphase onset [1]. Though our work shows that mRNAs for these components are provided maternally, the kinetics of checkpoint protein accumulation should be further investigated. Additionally, future work is required to determine the molecular differences between checkpoint signaling before and after the MBT to elucidate the molecular basis for the developmental timer.

## Materials and Methods

### Fish husbandry

Embryos were collected from natural mating and incubated in E3 buffer (5 mM NaCl, 0.17 mM KCl, 0.33 mM CaCl2, 0.33 mM MgSO4) at 28°C. All experiments were carried out in the Tuebingen long fin strain. The animal work performed in this study was approved by the University of Pennsylvania Institutional Animal Care and Use Committee. Adult animals were used only for mating and were not sacrificed. The NIH Policy on Humane Care and Use of Laboratory Animals applies to zebrafish embryos only after hatching, and all of our experiments were performed well before this stage.

### Cell cycle length measurements

For experiments using PCNA-GFP, recombinant protein was purified as described [23] from a GFP-fused human PCNA gene in pENeGFP-PCNA2 provided by Dr Michael Whitaker (Institute of Cell and Molecular Biosciences, University of Newcastle upon Tyne). Histone H1 from calf thymus (Sigma) was conjugated to Alexa-Fluor 647 (Invitrogen) following the manufacturer’s instructions. 1-cell stage embryos were injected with GFP-PCNA and AlexaFluor-tagged histone H1 and incubated in E3 buffer at 28°C until live imaging. For live imaging, embryos were dechorionated with 1 mg/mL pronase in E3 buffer for 10 min, washed 2x in E3 buffer, then mounted on a 4-well fluorodish (Grenier Bio-One) in 0.4% agarose dissolved in E3 buffer. For nocodazole-treated embryos, nocodazole was included in the E3 buffer to maintain the working concentration in the agarose. Images were acquired using a 20x 0.7 NA objective on an inverted fluorescence microscope (DM6000, Leica Microsystems) equipped with an automated XYZ stage and an electron multiplier charge-coupled device camera (QuantEM, 512 SC; Photometrics), controlled by Metamorph Software (MDS Analytical Technologies). Embryos were imaged every minute by fluorescence. At each time point a z-series of 10 images was collected at 10 μm intervals. Cell cycle lengths were measured by manually tracking shuttling of GFP-PCNA into and out of nuclei defined by AlexaFluor-tagged histone H1, using Metamorph and ImageJ software. Images are shown as maximal intensity projections of the confocal z-series.

For Fig. 1, embryos were imaged from ~2.5–3 HPF to measure the mitotic timing of the cleavage cycles. For Fig. 3A, embryos were imaged live starting from 1.5 HPF, at approximately the 4th-5th cleavage cycle. We measured cleavage cycles 5–10 and averaged multiple embryos to estimate the length of each cleavage cycle. For Fig. 3B, embryos were imaged from 2.25 to ~3 HPF (the usual time of the MBT), and cell cycle times were averaged over 2–3 cell cycles. For Fig. 4, embryos were imaged starting from 3.1 HPF for one cell cycle until ~3.5 HPF.

### Embryo drug treatments

Nocodazole was dissolved in DMSO for a stock solution and diluted in E3 buffer for a final working concentration of 0.125 ug/mL. For pH3 staining, 3.25 HPF post-MBT embryos were treated with nocodazole and incubated at 28°C for 45 minutes, then fixed with 4% formaldehyde in PBS. Control embryos were maintained in E3 buffer containing 0.025% DMSO.

To inhibit transcription, 2 nL of 1 mg/mL α-amanitin dissolved in ddH2O was injected into the cell of 1-cell stage embryos. Embryos were incubated in E3 buffer at 28°C until drug treatment with nocodazole.

### Immunofluorescence

Embryos were fixed with 4% paraformaldehyde in PBST (PBS with 0.1% Tween-20) overnight at 4°C, then manually dechorionated and dehydrated in 100% methanol overnight at -20°C. Embryos were rehydrated the next day sequentially with 75%, 50% and then 25% methanol in PBST (5 min each), then permeabilized with 100% acetone at -20°C for 7 min, then blocked with buffer containing 20% Heat-inactivated FBS, 20% Blocking reagent (Roche) and 1% DMSO in PBST for 1 hr at room temperature. Antibodies were diluted in blocking buffer, applied to embryos and incubated overnight at 4°C. Embryos were then washed four times (30 min each) with PBST, incubated with secondary antibody in blocking buffer, washed another three times, stained for 5 min with SYTOX Green (Invitrogen), then washed once with PBST. Embryos were mounted on fluorodishes (World Precision) in 4% methylcellulose dissolved in E3 buffer. Primary antibody was a mouse monoclonal against phospho-Ser10 Histone H3 (1:1000, Millipore cat #05–806). Secondary antibodies were Alexa-Fluor 647-conjugated anti-mouse or anti-rabbit (1:200, Invitrogen).

All fixed embryos were imaged with a spinning disk confocal: a microscope (DM4000, Leica) with a 20x 0.7 NA objective, a XY piezo-z stage (Applied Scientific Instrumentation), a spinning disk (Yokogawa), an electron multiplier charge-coupled device camera (ImageEM, Hamamatsu Photonics), and an LMM5 laser merge module equipped with 488 and 593 nm lasers (Spectral Applied Research) controlled by Metamorph software. Percentages of mitotic cells were quantified by counting pH3 positive nuclei as a fraction of total nuclei (based on DNA staining).

### Nuclear density measurements

Control and Chk1-4E embryos were imaged live as described above, and a z-projection of 10 images with 10 μm distance between slices was made for each embryo. We manually counted the number of nuclei within an isolated field measuring 118 x 118 μm for individual embryos for each condition, and used this number to represent the nuclear density.

### mRNA injections

Wildtype zebrafish Chk1 cDNA was purchased from ATCC (Cat no. 5410666) and cloned into a GFP-pCS2+ expression vector. The constitutively active, phosphomimetic zChk1 was created by mutating four residues (S256E, S280E, T292E, S301E) in zebrafish Chk1. These residues have been shown to be critical for Chk1 kinase activity [27]. The Ambion mMessage mMachine SP6 *in vitro* transcription kit was used to make Chk1-4E mRNA.

## Supporting Information

S1 Movie Live imaging of Histone H1 in an untreated embryo. The embryo was injected with Alexa 647-Histone H1 and PCNA-GFP proteins, corresponding to Fig. 1.
(AVI)

S2 Movie Live imaging of PCNA-GFP in an untreated embryo. The embryo was injected with Alexa 647-Histone H1 and PCNA-GFP proteins, corresponding to Fig. 1.
(AVI)

S3 Movie Live imaging of Histone H1 in a nocodazole treated embryo. The embryo was injected with Alexa 647-Histone H1 and PCNA-GFP proteins and treated with nocodazole, corresponding to Fig. 1.
(AVI)

S4 Movie Live imaging of PCNA-GFP in a nocodazole treated embryo. The embryo was injected with Alexa 647-Histone H1 and PCNA-GFP proteins and treated with nocodazole, corresponding to Fig. 1.
(AVI)
